# Toric Intraocular Lenses for the Management of Corneal Astigmatism at the Time of Cataract Surgery

**DOI:** 10.1155/2021/3286043

**Published:** 2021-12-18

**Authors:** Colm McAlinden, David Janicek

**Affiliations:** ^1^Department of Ophthalmology, Singleton Hospital, Swansea Bay University Health Board, Port Talbot, UK; ^2^Department of Ophthalmology, Royal Gwent Hospital, Aneurin Bevan University Health Board, Newport, UK

## Abstract

**Methods:**

This audit was conducted in a UK ophthalmology department and included 48 eyes of 42 patients. Surgery was performed during 2019 in patients with 2.50 diopters (D) or more corneal astigmatism. Anterior keratometry readings were used to determine the toric IOL power. Vector analysis using the Alpins method was used to assess changes in astigmatism pre to postoperatively.

**Results:**

There were 18 right and 26 left eyes included. In terms of gender, 61% of patients were female and 39% were male. The mean (±standard deviation (SD)) age was 70 (±11) years. The mean (±SD) axial length, *K*1, *K*2, and delta *K* was 23.55 (±1.4) mm, 42.71 (±1.39) D, 45.78 (±1.60) D, and 3.01 (±0.89) D, respectively. Postoperatively, the median spherical, cylinder, and spherical equivalent refraction was 0.00 D, −1.00 D, and 0.00 D, respectively. Postoperatively, 41% of the eyes had ≤0.50 D of spectacle astigmatism and 80% had ≤1.00 D. No patient required a secondary procedure to reposition the IOL from rotation. In vector analysis with the use of polar diagrams, there was a tendency for overcorrection of with-the-rule astigmatism and undercorrection of against-the-rule astigmatism.

**Conclusions:**

Significant reductions in astigmatism can be achieved with the use of toric IOLs in patients undergoing cataract surgery. Further improvements may be possible with surgeon-specific determination of their surgically induced astigmatism and flattening effect from the main corneal incision. Furthermore, the use of an optical biometer that directly measures the posterior corneal curvature and permits automatic toric IOL power determination with modern formulas avoiding the need for manual data entry may reduce the risk of human error and improve visual and refractive outcomes.

## 1. Introduction

Cataract surgery is one of the most commonly performed operations in the NHS [1]. Phacoemulsification is almost universally the technique employed to remove the cataract, and following this, an intraocular lens (IOL) is implanted into the remaining capsular bag [2]. The power of this lens is accurately determined with the use of ocular biometry, a device which chiefly measures the axial length of the eye and curvature of the cornea [3]. These parameters, as well as other parameters can then use via various formulas to determine the optimal IOL power for an individual eye for the desired refractive outcome [4].

In patients with astigmatism, where the refractive power of the eye is most in one direction and least 90 degrees away, a standard monofocal IOL is unable to correct both powers. In such situations, the residual astigmatism causes reduced uncorrected visual acuity [5]. Astigmatism may be corrected postoperatively with the use of spectacles or contact lenses. Alternatively, incisional techniques may be used at the time of cataract surgery to induce changes in the curvature of the cornea in an attempt to reduce corneal astigmatism. This is based on the coupling effect, a term to describe the ratio of the flattening of the principal (steeper) meridian to the steepening of the flatter meridian with corneal incisions [6]. The astigmatic effect with corneal incisions is influenced by many factors including incision size, location, tunnel length, patient age, corneal diameter, and corneal thickness. Techniques include limbal relaxing incisions, arcuate keratotomy, and opposite clear corneal incisions [7]. However, the amount of astigmatic correction possible with incisional techniques is low, may be unpredictable, and may induce irregular astigmatism [8]. In such cases, a toric IOL may be considered. Toric IOLs have a different power across the two principal meridians to correct corneal astigmatism. These lenses are ususally specifically ordered on an individual patient basis and require additional surgical steps [9].

The purpose of this quality improvement project was to assess astigmatic outcomes with the use of toric IOLs for patients with significant amounts of corneal astigmatism undergoing cataract surgery. Furthermore, we aimed to analyze these outcomes using vector analysis in an attempt to make recommendations to help improve outcomes.

## 2. Methods

In our NHS ophthalmology department based at Singleton Hospital, Swansea, toric IOLs are offered to patients with 2.50 diopters (D) or more corneal astigmatism. If patients have more than 4.00 D of corneal astigmatism, they undergo corneal topography to exclude corneal diseases which are known to induce high levels of astigmatism such as keratoconus [10]. Patients with the following conditions are generally excluded for toric IOLs: corneal ectasia, pterygium, Fuchs endothelial dystrophy, lens subluxation/dislocation, severe amblyopia, or significant general health comorbidity.

Prior to ocular biometry which allows determination of the IOL power required, patients who wore contact lenses were advised to avoid use for 1 week or 2 weeks if soft or hard contact lenses were used, respectively. The IOLMaster 700 or 500 (Car Zeiss Meditec, Germany) was used for biometry. In cases of keratometry readings (K-readings) less than 41 D or greater than 47 D, previous laser refractive surgery, irregular corneas, astigmatism greater than 4.00 D, or keratometry not possible with the IOLMaster, the Pentacam tomographer (Oculus, Germany) was used to further investigate suitability for a toric IOL, and if suitable, the K-readings were taken from the Pentacam in the calculation of the necessary toric IOL power.

All patients suitable for a toric IOL had the SN6AT AcrySof IQ toric (Alcon, USA) implanted at the time of surgery. The Alcon online toric IOL calculator incorporating the Barrett toric calculator was used to determine the required IOL power. This lens is biconvex with an aspheric anterior surface and a toric posterior surface. It also has ultraviolet and blue light filtering properties. It is made from a hydrophobic acrylic material with a refractive index of 1.55. Immediately prior to cataract surgery, all patients have their corneas marked at the slit lamp to reduce potential errors from cyclorotation when moving from the upright to supine position. During surgery, the toric IOL was implanted into the capsular bag and rotated into the desired axis. The main incision size varied between 2.2 mm and 2.8 mm among the different surgeons with a superior or superior temporal location. All patients are seen at one day postoperatively to check axis alignment.

We conducted an audit of all toric IOLs implanted during the year 2019 in our surgical centre. Outcomes were assessed by comparison of preoperative anterior corneal K-readings to postoperative spectacle refraction including astigmatism. The purpose of a clinical audit is to compare clinical practice to standards as part of clinical governance. The audit criterion is an explicit statement defining an outcome to be measured [11]. The criterion was determined from the toric IOL results published in a Cochrane review by Lake and colleagues [12] who reported that 70% of the eyes had 0.50 D or less astigmatism postoperatively.

## 3. Results

In total, 48 eyes of 42 patients were included (42 unilateral and 6 bilateral). A total of 8 surgeons performed the surgeries. Four eyes were excluded from the analysis due to 2 eyes received a different toric IOL manufacturer (as outside the power range for the SN6ATT AcrySof IQ Toric lens) and 2 eyes had missing pre or postoperative data. The Barrett toric formula was used to calculate the required IOL power using the anterior K-readings [13].

A total of 18 right and 26 left eyes were included. In terms of gender, 61% of patients were female and 39% were male. The mean (±standard deviation (SD)) age was 70 (±11) years. The mean (±SD) axial length, *K*1, *K*2, and delta *K* was 23.55 (±1.4) mm, 42.71 (±1.39) D, 45.78 (±1.60) D, and 3.01 (±0.89) D, respectively. The mean (±SD) target refraction in spherical equivalent (SE) and residual cylinder was −0.08 (±0.16) D and 0.16 (±0.13) D, respectively.

In terms of postoperative outcomes, the mean (±SD) spherical, cylinder, and SE refraction was 0.09 (±0.56) D, −0.78 (±0.69) D, and −0.30 (±0.50) D, respectively. The median values for these parameters were 0.00 D, −1.00 D, and 0.00 D, respectively. No patient required a secondary procedure to reposition the IOL due to rotation.

**Figure 1:**
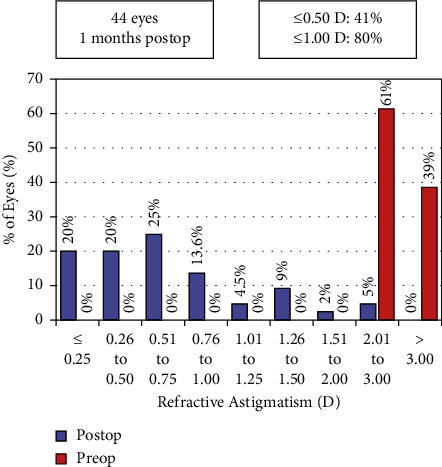
Pre and postoperative refractive astigmatism by percent.

**Figure 2:**
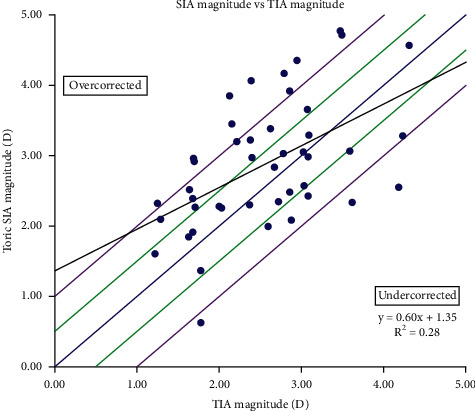
A plot of the surgically induced astigmatism (SIA) magnitude versus the target-induced astigmatism (TIA) magnitude, demonstrating a slight tendency for undercorrection.

**Figure 3:**
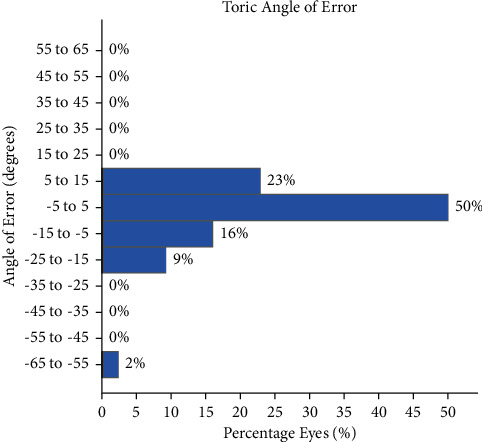
Assessment of the toric angle of error with 48% of the eyes within ±5 degrees, 20% between 5 and 15 degrees of counterclockwise rotation and the remainder rotated clockwise.

**Figure 4:**
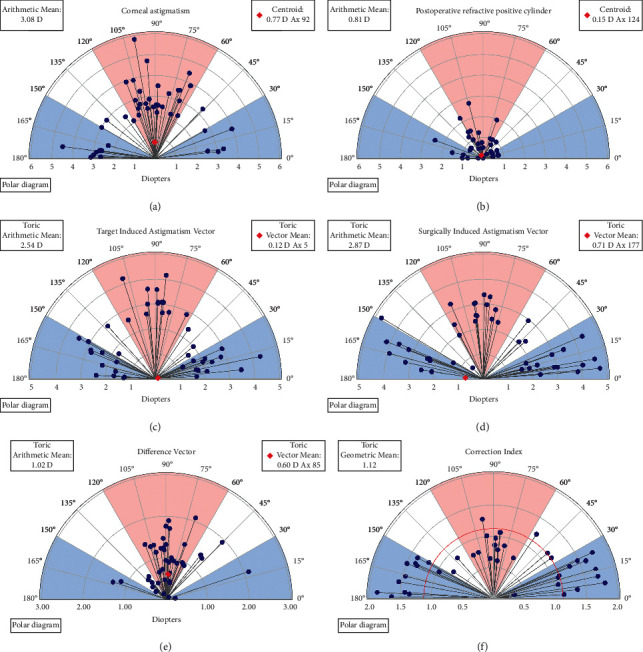
Polar diagrams showing the preoperative corneal astigmatism (a), postoperative refractive positive cylinder (b), target-induced astigmatism vector (c), surgically induced astigmatism vector (d), difference vector (e), and correction index (f). These polar diagrams demonstrate a tendency for overcorrection of with-the-rule astigmatism and undercorrection for against-the-rule astigmatism.

Figure 1 shows the refractive outcomes showing 41% of the eyes had ≤0.50 D of astigmatism and 80% had ≤1.00 D. The Alpins astigmatism analysis [14] was used to further investigate astigmatic refractive outcomes. This methodology stems around three vectors: the target induced astigmatism vector (TIA), surgically induced astigmatism vector (SIA), and the difference vector (DV). Definitions of these terms and their formulas have been previously reported [14].  Figure 2 shows a plot of the SIA magnitude versus the TIA magnitude, with a slight tendency for undercorrection.  Figure 3 shows the assessment of the toric angle of error with 50% of the eyes within ±5 degrees, 23% between 5 and 15 degrees of counterclockwise rotation, and the remainder rotated clockwise.  Figure 4 shows an array of polar diagrams showing the preoperative corneal astigmatism, postoperative refractive positive cylinder, target-induced astigmatism vector, surgically induced astigmatism vector, difference vector, and correction index. These polar diagrams demonstrate a tendency for overcorrection of with-the-rule astigmatism and undercorrection for against-the-rule astigmatism.

## 4. Discussion

This quality improvement project looked at the outcomes of toric IOLs used at the time of cataract surgery in patients with significant preoperative corneal astigmatism. The preoperative corneal astigmatism for the group was approximately 3 diopters which was reduced to a spectacle astigmatism of 0.50 diopters or less astigmatism in 41% of patients and 1.00 diopter or less in 80%. Hence, we did not meet the audit criterion set at 70% with less than 0.50 diopters of astigmatism. However, it must be remembered that the figure of 70% was taken from a large Cochrane review of randomized controlled trials (RCTs), and it is well known that there tends to be a mismatch between real-world and RCT outcomes. The strict inclusion and exclusion criteria with trial populations are often not representative of the populations of patients and other variables encountered in routine clinical practice [15].

With the further analysis using the Alpins astigmatism vector method, we identified a slight tendency for clockwise rotation from the intended axis. We also identified a tendency for overcorrection of with-the-rule astigmatism, and on the contrary, a tendency for undercorrection of against-the-rule astigmatism. The vectorial value of ocular residual astigmatism (ORA) most commonly has an orientation of close to 180 degrees, much of this contributed by the posterior corneal power. So, measurement of the total corneal power (TCP) from the Oculus Pentacam and use of this for the corneal astigmatism value in biometry formulas, instead of only the anterior K-readings, would have had the net effect of reducing the prevailing overcorrection of with-the-rule astigmatism and increasing the correction index of against-the-rule corneas that are in the first polar diagram in Figure 4. There were some cases with large angles of error. However, it must be considered that the online Alcon toric IOL calculator recommended a “flipped” axis (by 90 degrees) for some cases to result in a lower residual cylinder power. With low amounts of postoperative astigmatism, the endpoint of subjective refraction can be “soft” with a wide range of visually acceptable axes for the patient. There is also the precision of the refraction technique performed by the community optometrist to consider.

To better interpret our results, we need to consider the limitations of the study. First, 6 bilateral cases were included, which on the one hand helps increase the sample size; it has the disadvantage that the two eyes of one patient tend to be more correlated than two eyes from two patients; hence, they may not be considered statistically independent variables [16]. There were also 8 different surgeons who can induce errors due to differing techniques for marking the cornea preoperatively and differing surgical techniques. The online toric IOL calculator requires the preassessment nursing staff to manually enter patient variables which increase the possibility of typographical errors. Another limitation is that the toric IOL is only available in 0.50–0.75 D steps which reduces precision. Furthermore, multiple biometers were used, and there may be differences in the precision between devices [17]. There are also the errors in other measurements such as postoperative refraction performed by the patient's community optometrist, and also, there were a number of different optometrists providing these data. However, it must be remembered that this is a real-world evaluation and not an RCT, and despite these limitations, the analysis provides valuable information on toric IOL outcomes and permits recommendations in an attempt to improve clinical outcomes and ultimately improve patient quality of life.

Considering the results and noting the limitations, we have made recommendations within our department to help improve toric IOL outcomes. First, we will aim to use the latest version of the IOLMaster 700 (Carl Zeiss Meditec) rather than the previous version (IOLMaster 500) as it permits direct measurement of the posterior corneal curvature [18]. With the direct measurement of posterior corneal curvature, more modern toric IOL formulas can be used which may improve refractive outcomes, as has been shown in other studies [19]. This can all be performed directly on the biometer and avoids the need for the preassessment nurse to navigate to the online calculator, which only considers anterior corneal curvature measurements to be entered. As the values need to be manually inputted to the online calculator, this increases the risk of typographical errors.

The other consideration is the determination of surgeon-specific surgically induced astigmatism (SIA), that is, the astigmatism induced by the main corneal incision. This can be easily determined by comparing pre to postoperative corneal astigmatism power and axis in a cohort of patients. Online calculators are available to allow the mean SIA to be calculated for a cohort of patients for an individual surgeon, which can then be incorporated into prospective toric IOL power calculations. Furthermore, the use of the flattening effect (FE) rather than the SIA is a more accurate means of including incisional behaviour avoiding overestimation of the incisional effect and systematic undercorrection of the toric implant power chosen. Other considerations include the use of the Zeiss Callisto or Alcon Verizon system to project for markerless toric IOL alignment, hence reducing errors. The use of TCP rather than anterior K-readings only, such as those obtained from the Oculus Pentacam, would provide a composite of the anterior and posterior corneal curvature. This would then permit the use of a toric calculator that has the facility of utilizing total corneal power in its data entry. TCP measurement may be more useful in a calculator that accommodates this parameter for calculation of the necessary toric power rather than a separate measurement of posterior corneal power which by itself with available calculators is not easily included in the toric power calculation.

## 5. Conclusions

Significant reductions in ocular astigmatism can be achieved with the use of toric IOLs at the time of cataract surgery. Although results can be improved, moving to the exclusive use of an optical biometer which also measures posterior corneal curvature as well as automatic calculation of the toric IOL power needed is desirable. This avoids the need for manual data entry to an online calculator. Individual surgeon-specific SIA and FE calculations can also be considered in an attempt to further improve the precision of astigmatism management with toric IOLs.

## Data Availability

The data used to support the findings of this study are available from the corresponding author upon request.
